# Venereal Prevention

**Published:** 1920-07-31

**Authors:** Ali Baba

**Affiliations:** 218 Wellhall Road, Eltham, Kent


*To the Editor of* THE HOSPITAL.

### Venereal Prevention.

*To the Editor of* THE HOSPITAL.

SIR,—I was much interested in the letter you published recently by "Social Reformer." For a considerable time I have been attracted by the various opinions which have appeared in the Press on the different means of combating and preventing venereal disease. It is a subject which certainly has a curious fascination for students of sociology, and it is not until the different points of view are well probed that the vital importance of the question appears. To my mind it is necessary to work towards an "ideal" in all subjects of social reform, and, therefore, in the case of fighting venereal disease the "ideal" must be the *total abolition* of venereal disease in the country. As stepping-stones to this such legislation as compulsory notification, more stringent control of the sale of alcohol, and amendments to the criminal law are probably necessary, but the outstanding feature of all is the importance of raising the standard of public morality.

Would this be attained if "packets" were sold in every chemist's shop, inviting promiscuous sexual intercourse? Is such an ideal in the mind of the Society for Prevention of Venereal Disease when it issues these leaflets of "directions for men" and "directions for women"?—the most morally degrading pamphlets I have ever seen issued by a body of eminent men. I wonder if any more of your readers have read "Social Reformer's" letter in connection with the paragraph you published in the same issue of THE HOSPITAL under the heading of "Social Welfare," giving a synopsis of a part of the work of the National Council for Combating Venereal Disease.—I am, yours faithfully,

ALI BABA.

218 Wellhall Road, Eltham, Kent, July 20, 1920.

